# Supplementary figures and images for: The Transcription Factor EB Reduces the Intraneuronal Accumulation of the Beta-Secretase-Derived APP Fragment C99 in Cellular and Mouse Alzheimer’s Disease Models
Source: Cells. 2020 May 12;9(5):1204. doi: 10.3390/cells9051204 (PMC7291113; doi:10.3390/cells9051204)

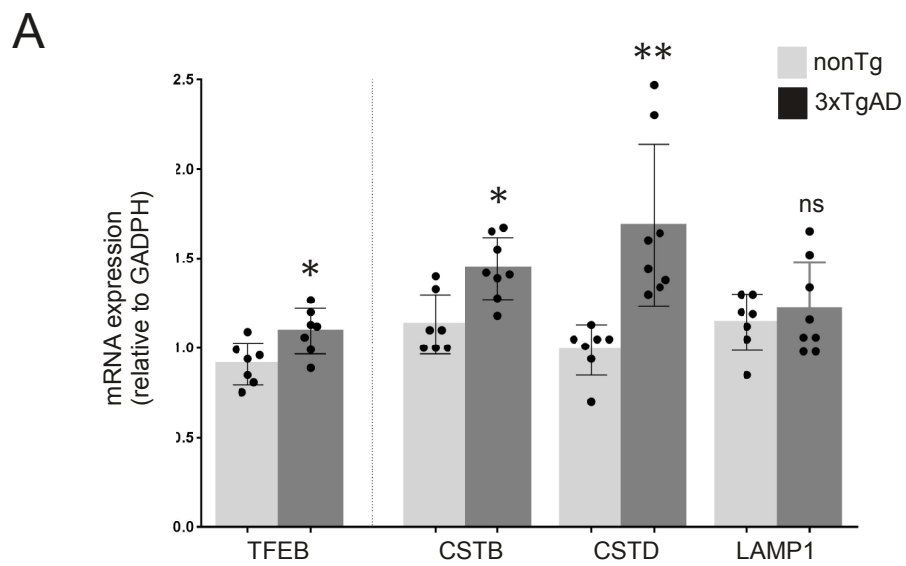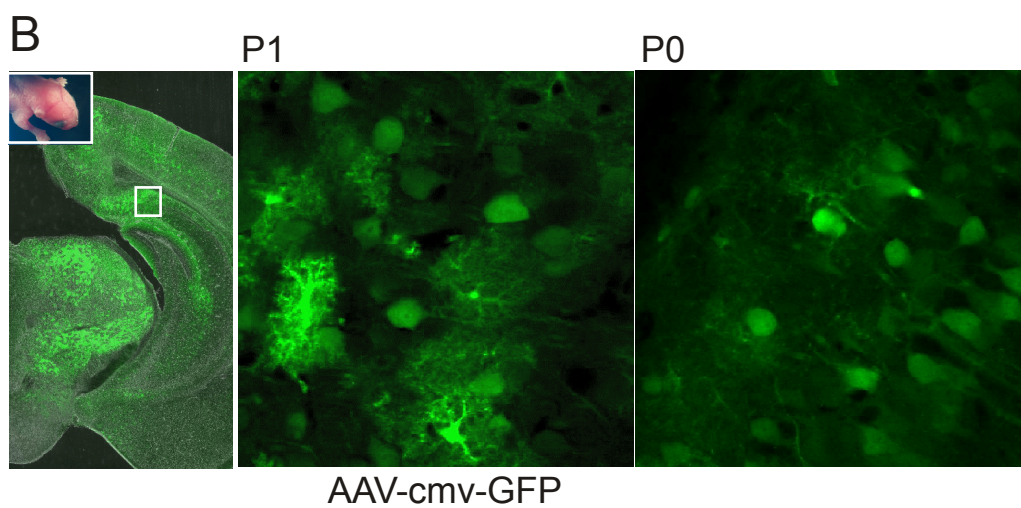

Suppl. Figure 1

Supplement: Supplementary file 1 [file cells-09-01204-s001.pdf]
